# Alterations of the gut microbiota associated with the occurrence and progression of viral hepatitis

**DOI:** 10.3389/fcimb.2023.1119875

**Published:** 2023-06-05

**Authors:** Xing Yang, Huanzhuo Mai, Jie Zhou, Zhuoxin Li, Qing Wang, Liuyan Lan, Fang Lu, Xiping Yang, Baodong Guo, Li Ye, Ping Cui, Hao Liang, Jiegang Huang

**Affiliations:** ^1^ School of Public Health, Guangxi Medical University, Nanning, China; ^2^ Guangxi Key Laboratory of AIDS Prevention and Treatment, Guangxi Universities Key Laboratory of Prevention and Control of Highly Prevalent Disease, Guangxi Medical University, Nanning, China; ^3^ Life Science Institute, Guangxi Medical University, Nanning, China; ^4^ Collaborative Innovation Centre of Regenerative Medicine and Medical BioResource Development and Application Co-constructed by the Province and Ministry, Guangxi Medical University, Nanning, China; ^5^ Guangxi Colleges and Universities Key Laboratory of Prevention and Control of Highly Prevalent Diseases, Guangxi Medical University, Nanning, China

**Keywords:** gut microbiota, 16s ribosomal RNA gene amplicon sequencing, viral hepatitis, liver disease, microbial markers

## Abstract

**Background:**

Gut microbiota is the largest population of microorganisms and is closely related to health. Many studies have explored changes in gut microbiota in viral hepatitis. However, the correlation between gut microbiota and the occurrence and progression of viral hepatitis has not been fully clarified.

**Methods:**

PubMed and BioProject databases were searched for studies about viral hepatitis disease and 16S rRNA gene sequencing of gut microbiota up to January 2023. With bioinformatics analyses, we explored changes in microbial diversity of viral hepatitis, screened out crucial bacteria and microbial functions related to viral hepatitis, and identified the potential microbial markers for predicting risks for the occurrence and progression of viral hepatitis based on ROC analysis.

**Results:**

Of the 1389 records identified, 13 studies met the inclusion criteria, with 950 individuals including 656 patient samples (HBV, *n* = 546; HCV, *n* = 86; HEV, *n* = 24) and 294 healthy controls. Gut microbial diversity is significantly decreased as the infection and progression of viral hepatitis. Alpha diversity and microbiota including *Butyricimonas*, *Escherichia-Shigella*, *Lactobacillus*, and *Veillonella* were identified as the potential microbial markers for predicting the risk of development of viral hepatitis (AUC>0.7). Microbial functions including tryptophan metabolism, fatty acid biosynthesis, lipopolysaccharide biosynthesis, and lipid metabolism related to the microbial community increased significantly as the development of viral hepatitis.

**Conclusions:**

This study demonstrated comprehensively the gut microbiota characteristics in viral hepatitis, screened out crucial microbial functions related to viral hepatitis, and identified the potential microbial markers for predicting the risk of viral hepatitis.

## 1 Introduction

Viral hepatitis is a significant healthcare burden worldwide, resulting in around 300 million infections and more than 1.3 million deaths each year (Policy, 2017). It is caused by five types of viruses: hepatitis A virus (HAV), hepatitis B virus (HBV), hepatitis C virus (HCV), hepatitis D virus (HDV), and hepatitis E virus (HEV). HBV and HCV are responsible for about 90% of the mortality from viral hepatitis (Jefferies et al., 2018), and long-term infection could lead to other dangerous consequences such as liver cirrhosis (LC), hepatocellular carcinoma (HCC), and even death (Winer et al., 2017; Datfar et al., 2021; Su et al., 2022b). HDV is an incomplete virus that requires HBV for its replication and globally affects around 5% of people with HBV infection(WHO). HAV and HEV are the common infectious etiologies of acute hepatitis throughout the world (Lin et al., 2017; Aslan and Balaban, 2020). Given the great threat of viral hepatitis to public health, it is meaningful to explore how to reverse hepatitis virus infection and to prevent the development of end-stage liver disease.

Researchers have proved that the immune system is involved in one way or another in patients with viral hepatitis (Barathan et al., 2018; Su et al., 2023). It could control the viral infection; however, the long-lasting inflammation could lead to hepatic damage (Hou et al., 2018). Thus, the immune responses are critical for determining the outcome of hepatitis virus infection (Su et al., 2022a; Chi et al., 2023). The gut microbiota is the largest population of microorganisms in the human body. It resides in the intestine and is made up of trillions of bacteria, fungi, and other microbes. It is critical for the host defense and immune homeostasis in humans (Rinninella et al., 2019; Xue et al., 2020). Alterations in the gut microbiota are associated with immune homeostasis disturbances (Yang and Cong, 2021). Recent studies have raised the possibility that gut microbial dysbiosis is involved in the occurrence and progression of viral hepatitis (Chen et al., 2022; Ali et al., 2023).

Hepatitis virus infection alters the diversity of the gut microbiome. One study showed increased diversity of microbiota in the treatment-naive HCV group (Sultan et al., 2021), while another study showed the opposite result, with lower alpha diversity among HCV-infected patients (Aly et al., 2016). Other researchers have found significant changes in the gut microbiome among HBV-induced disease groups when compared with healthy individuals. For example, the abundance of *Veilonella*, *Streptococcus*, and *Enterococcus* increased significantly in the HBV-related acute-on-chronic liver failure (HBV-ACLF) group, and *Bacteroides*, *Lachnospiracea incertae sedis*, and *Clostridium* cluster XIVa were enriched in patients with HBV-related HCC patients (HBV-HCC) (Yang et al., 2018; Huang et al., 2020; Yao et al., 2021). Nevertheless, it is not completely clear whether there is a correlation between the microbiome change in different types of viral hepatitis. How the gut microbiome is altered during the process from hepatitis virus infection to chronic hepatic disorders needs to be studied in greater detail. Besides, how alterations in the gut microbiota affect the development of viral hepatitis is not well understood.

Therefore, we conducted this study using available data of 16S ribosomal RNA (rRNA) gene amplicon sequencing of gut microbiota-related viral hepatitis disease studies. We reanalyzed these data by using rigorous bioinformatics methods to elucidate shifts in the microbial community structure and diversity along with the development of viral hepatitis and to reveal crucial microbial taxonomy and functions related to viral hepatitis, aiming to provide recommendations for delaying disease progression during hepatic exacerbation.

## 2 Materials and methods

### 2.1 Search strategy and selection criteria

The PubMed (https://pubmed.ncbi.nlm.nih.gov/) and BioProject (https://www.ncbi.nlm.nih.gov/bioproject) databases were searched for articles and data about viral hepatitis and the gut microbiota published up to January 2023. The search strategy was developed based on keywords, medical subject headings (MeSH) terms, and synonyms (**Table S1**). Studies were included according to the following criteria: (1) it included a viral hepatitis or its related hepatic disease group and a control group that can be distinguished, (2) it used stool or rectal swab samples used for sequencing, and (3) it employed 16S ribosomal RNA (16S rRNA) gene sequencing and the data available. Raw 16S rRNA gene sequence and metadata information were downloaded from publicly available databases or obtained from the authors. Animal experiments or *in vitro* studies, reviews, meta-analyses, comments, letters, poster abstracts, and studies with less than three individuals (either case or control groups) were excluded.

### 2.2 Processing of raw data

The Sequence Read Archive (SRA) files were downloaded from the National Center for Biotechnology Information (NCBI) SRA database and converted to FASTQ format by the SRA Toolkit (https://github.com/ncbi/sratoolkit, Version 3.0.0). These files were then imported into Quantitative Insights in Microbial Ecology (QIIME) version 2 for microbiome bioinformatics analysis(Estaki et al., 2020). The QIIME2 DADA2 plug-in, which takes input dereplicated amplicon sequencing reads as an input, removed the primers and low-quality reads from the sequences, and finally returned the inferred composition of the samples. This information was used to implement sequence quality control for single-end and paired-end 16S rRNA gene reads(Callahan et al., 2016; Estaki et al., 2020). The outputs of the DADA2 pipeline were representative sequences, some statistics on the procedure, and a feature table of amplicon sequence variants (ASVs) with ≥ 97% identity matches to the expected sequences in the extreme dataset(Nearing et al., 2018).

### 2.3 Filtering data

After generating an ASV table and representative sequences, a q2-feature-table plug-in in QIIME2 was used to filter the low-quality features to improve the quality of the downstream statistical analysis. The samples containing the minimum count of sequences that might be caused by sequencing errors or a low level of the microbiome leading to low uptake of deoxyribonucleic acid (DNA) were filtered. Meanwhile, the low abundance features were filtered based on the interquartile range (IQR). Then the generated filtered sequences were classified with the q2-feature-classifier plug-in based on the SILVA database (https://www.arb-silva.de/, version 138) released in the QIIME2 package(Bokulich et al., 2018).

### 2.4 Data analysis

Files of the filtered feature table, phylogenetic tree, and taxonomic classifications were imported into RStudio 4.2.1. The vegan package was utilized to normalize the feature table to scale based on each sample’s library size that transformed the feature table into a relative feature table, aiming to remove technical bias caused by variations in sample collection, library preparation, or sequencing manifesting as uneven sampling depth and sparsity, which could not reflect the true difference in the underlying biology(Weiss et al., 2017). Then, the microeco package was used to calculate the alpha diversity indices including richness (Observed Species, Chao1, and ACE), diversity (Shannon, Simpson, Invsimpson, and Fisher), and phylogenetic diversity (PD) to evaluate the overall structure of the gut microbiota. Beta diversity was analyzed with the vegan package, based on principal coordinates (PCoA) and the non-metric multidimensional scaling (NMDS) analyses. Objects that are ordinated closer together have smaller dissimilarity values than those ordinated further farther apart. Next, the permutational multivariate analysis of variance (PERMANOVA) test and analysis of similarities (ANOSIM) based on the Bray-Curtis distance were performed to evaluate the similarities between the groups. Besides, based on the distribution of ASVs, the UpSet plot was used to show the ASV intersections among different groups through the UpSet package.

The SpiecEasi package was employed to identify the different genus and species through the linear discriminant analysis effect size (LEfSe) method (Linear Discriminant Analysis [LDA] score ≥2). The Kyoto Encyclopedia of Genes and Genomes (KEGG) functional pathways related to the microbial community were predicted with the Tax4Fun2 package based on 16S rRNA gene sequencing data classified with the SILVA database. Finally, the area under the curve (AUC) of the receiver operating characteristic (ROC) curve was calculated to evaluate the prediction effectiveness of the alpha diversity indices and differential microbial taxonomy.

The forest plots of the comparisons of the alpha diversity between the case and control groups were generated in Review Manager 5.3. A fixed-effects or random-effects model was selected according to the deviance information criterion (DIC). The standardized mean difference (SMD) and the corresponding 95% credible interval (CI) were used to evaluate the results. The beta diversity and LEfSe analyses were visualized by R 4.1.1. Graphs of the differential KEGG functional pathways were generated in GraphPad Prism 8.0.2. ROC curve analysis was conducted in GraphPad Prism 8.0.2. A model with an AUC score of > 0.7 would be considered acceptable. All tests were two-sided with a *P*-value of 0.05 set as the threshold for significance.

## 3 Results

### 3.1 Study characteristics

**Figure 1:**
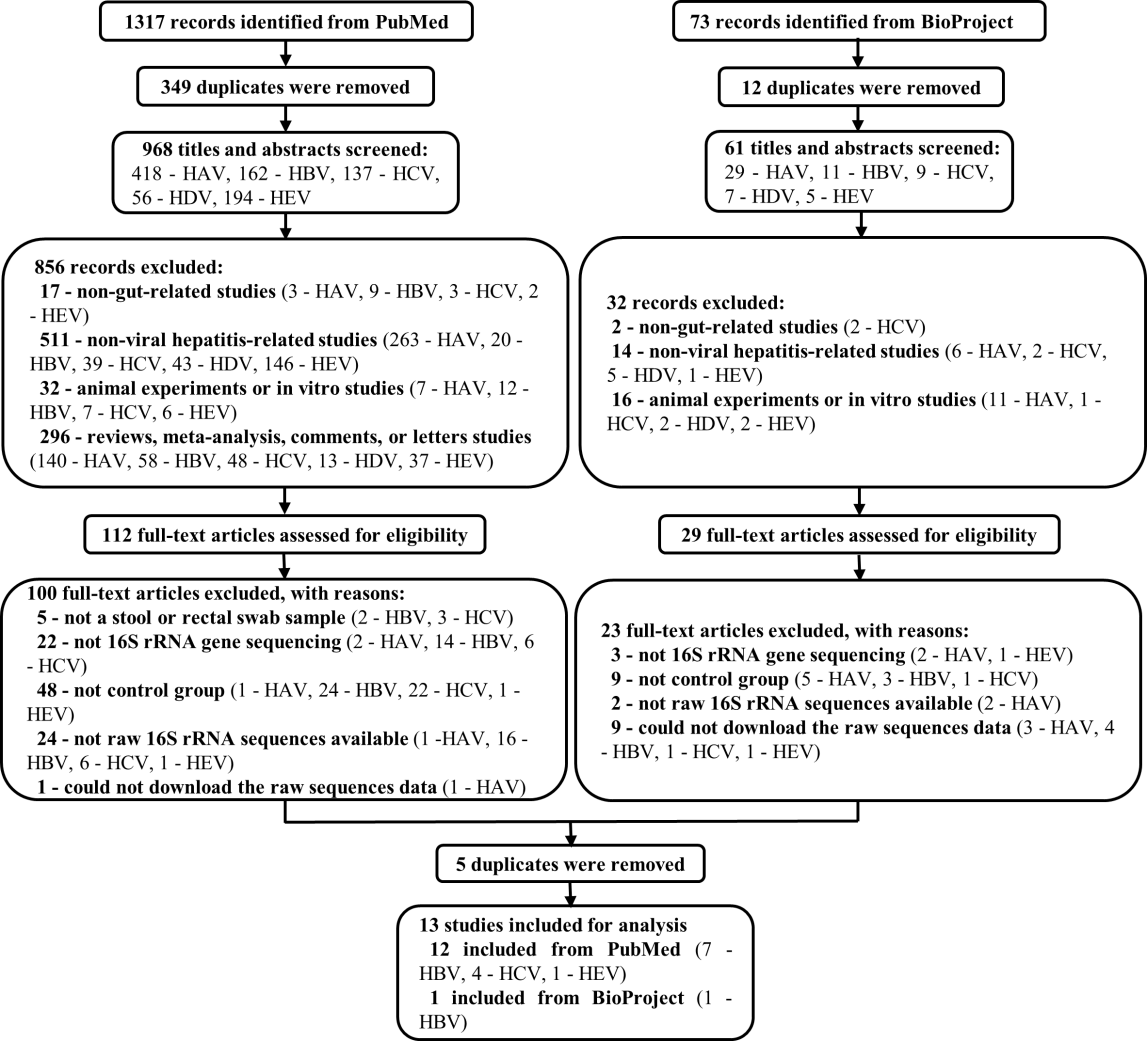
Flow chart of the study selection procedure. 1317 and 73 records were identified from PubMed and BioProgect, respectively. After removing the duplicates, 1029 titles and abstracts were screened. Studies that were non-gut-related studies, non-viral hepatitis-related studies, animal or *in vitro* studies, and reviews or meta-analyses were excluded. Then 141 full-text articles were assessed to exclude articles without a stool sample, 16S rRNA gene sequencing, or control groups. Finally, 13 studies were included for analysis, with a total of 950 individuals (HBV, *n* = 546; HCV, *n* = 86; HEV, *n* = 24; HC, *n* = 294).

Of the 1,317 studies found in PubMed and the 73 records found in BioProject, 13 records related to HBV, HCV, and HEV were eventually included for subsequent analysis. Note that all the records concerning HAV and HDV were excluded (**Figure 1**). Among the 12 included studies from PubMed, 1 was about HEV infection(Wu et al., 2020), 4 were about HCV infection(Aly et al., 2016; Taylor et al., 2020; Sultan et al., 2021; Ali et al., 2023), and 7 were HBV-related liver disorders (Wang et al., 2017; Liu et al., 2018; Liu et al., 2019; Ni et al., 2019; Chen et al., 2020; Zheng et al., 2020; Li et al., 2022). The remaining unpublished data was from BioProject and concerned HBV (BioProject accession number PRJEB32568). These studies were from China, the United States, and Egypt, and contained a total of 950 individual samples (HBV, *n* = 546; HCV, *n* = 86; HEV, *n* = 24; healthy control [HC], *n* = 294). More comprehensive details of the included studies are presented in **Table 1**.

**Table 1:** Study characteristics of the included studies.

Author, Year [Ref]	PMID	BioProject accession number	Country	Study settings	Study period	Sample size	Sample type	16S rRNA Variable Region, Sequencing Platform, Sequencing technology
Wu et al., 2020	32500937	ERP119119	China	The First Affiliated Hospital (College of Medicine) of Zhejiang University	2018.05-2019.05	12 HEV, 12 HEV-ALF	Stool samples	V3-V5, Ion Torrent S5 XL, Single read sequencing
Taylor et al., 2020	33051377	ERP122366	the United States	The University of California San Diego	NA	111 HC, 13 HCV	Stool samples	V4, Illumina MiSeq, Single read sequencing
Liu et al., 2019	30675188	PRJNA428932	China	Nanjing Medical University Affiliated Cancer Hospital	2016.09-2017.05	33 HC, 35 HBV-HCC	Stool samples	V4, Illumina HiSeq 2500, Single read sequencing
NA	NA	PRJEB32568	China	NA	NA	5 HC, 12 CHB, 11 HBV-LC, 9 HBV-HCC	Stool samples	NA, Illumina HiSeq 2500, Single read sequencing
Aly et al., 2016	27625705	PRJNA328966	Egypt	Faculty of Pharmacy, Cairo University	2015.02-2016.09	8 HC, 6 HCV	Stool samples	V4, Illumina MiSeq, Paired-end sequencing
Wang et al., 2017	29180991	PRJNA382861	China	The affiliated hospital of Shanghai University of Traditional Chinese Medicine, The Infectious Disease Hospital of Ningbo, and the Sixth of People’s Hospital of Shaoxing Zhejiang	NA	25 HC, 206 HBV	Stool samples	V3-V4, Illumina MiSeq, Paired-end sequencing
Chen et al., 2020	32265857	PRJNA558158	China	Zhongshan Hospital, affiliated with Xiamen University	2017.12-2018.05	21 HC, 23 HBV, 28 CHB, 25 HBV-LC	Stool samples	V3-V4, Illumina HiSeq 2500, Paired-end sequencing
Sultan et al., 2021	33119247	PRJNA634402	Egypt	Department of Endemic Hepatology and Gastroenterology, Mansoura University Hospitals	NA	38 HC, 38 HCV	Stool samples	V3-V4, Illumina MiSeq, Paired-end sequencing
Liu et al., 2018	29780327	PRJNA445763	China	the First Affiliated Hospital of Harbin Medical University	2015.12-2016.12	20 HC, 28 HBV-LC	Stool samples	V3-V4, Illumina HiSeq 2500, Paired-end sequencing
Ni et al., 2019	31293562	PRJNA478823	China	Zhujiang Hospital, Southern Medical University	2017.08-2017.11	18 HC, 61 HBV-HCC	Stool samples	V4-V5, Illumina MiSeq, Paired-end sequencing
Zheng et al., 2020	32281295	PRJNA540574	China	the First Hospital of Jilin University	2017.03-2018.04	20 HC, 8 CHB, 35 HBV-HCC	Stool samples	V4, Illumina HiSeq 2500, Paired-end sequencing
Ali et al., 2023	36522461	PRJNA727609	the United States	the National Institutes of Health Clinical Center	2015.06-2017.02	13 HCV, 16 HCV-LC	Stool samples	V4, Illumina MiSeq, Paired-end sequencing
Li et al., 2022	35733959	PRJNA838083	China	Guangzhou Panyu Central Hospital	2020.10-2021.7	15 HC, 23 CHB, 20 HBV-LC, 22 HBV-HCC	Stool samples	V4, Illumina Nova6000, Paired-end sequencing

NA, Not Applicable.

### 3.2 Hepatitis virus infection and progression significantly reduce gut microbial diversity

**Figure 2:**
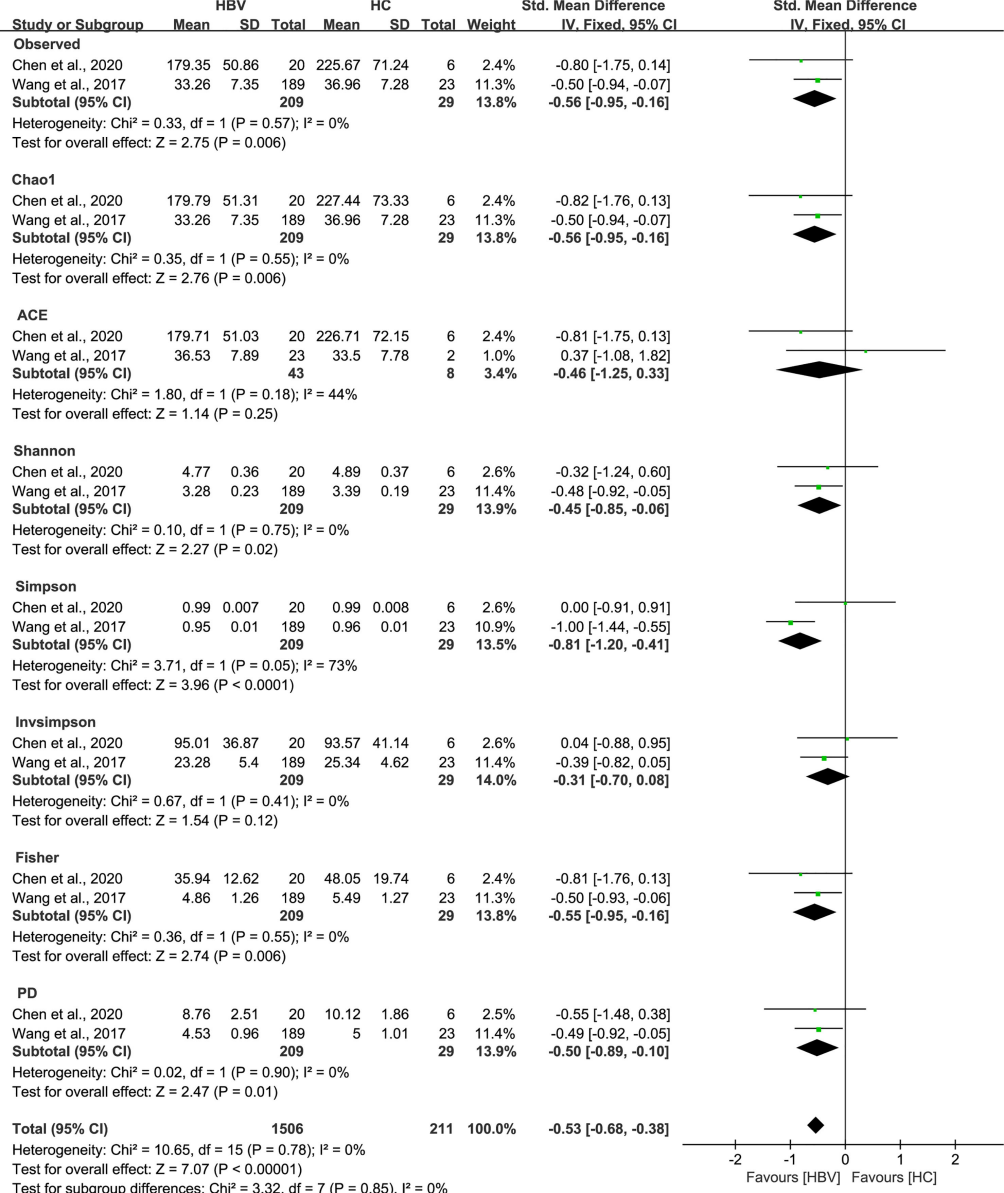
The gut microbial alpha diversity of patients infected with HBV was decreased. As estimated by the Observed, Chao1, Shannon, Simpson, Fisher, and PD indexes, the alpha diversity reduced significantly in HBV-infected patients (n=209) compared with healthy individuals (n=29).

**Figure 3:**
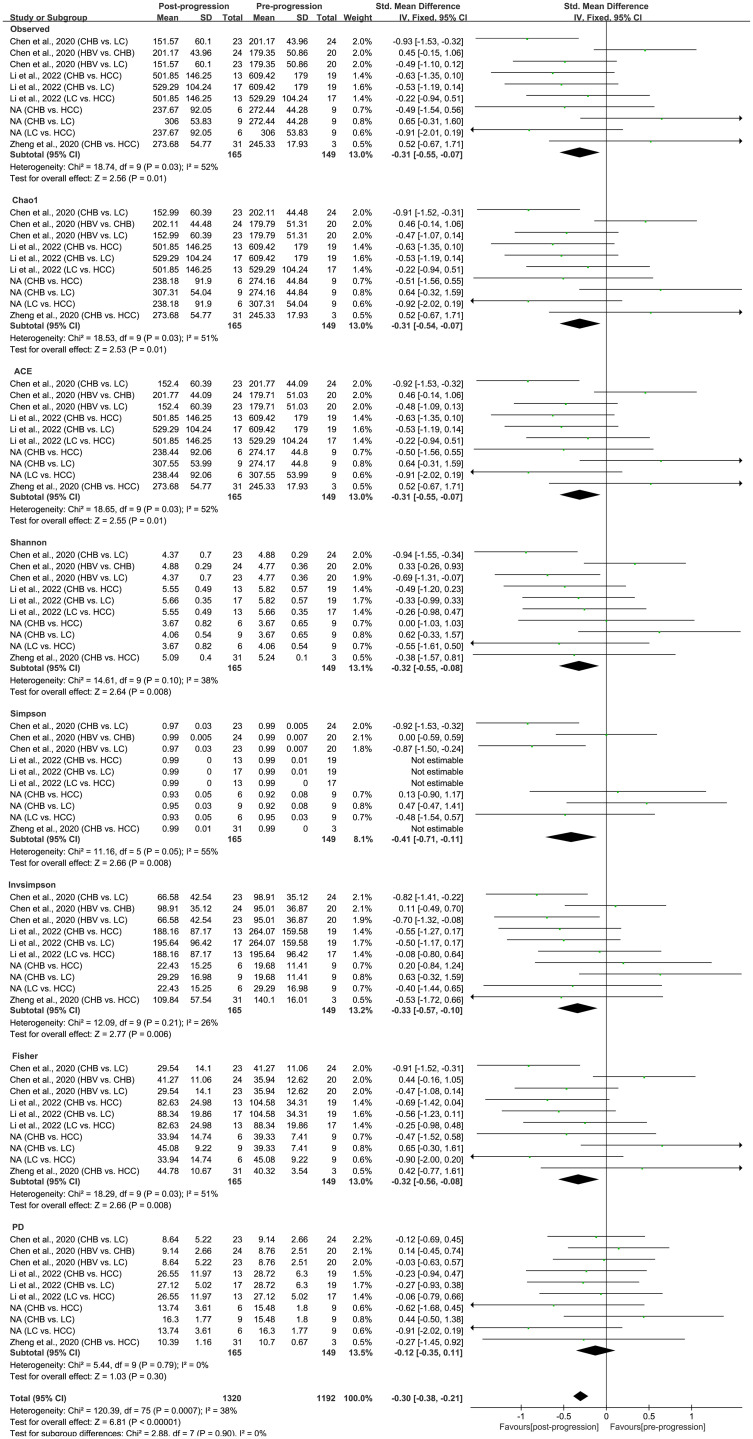
The gut microbial alpha diversity decreased with the progression of HBV infection. During the period of HBV progression, there exist different phases of disease including HBV infection, CHB, HBV-LC, and HBV-HCC. “Pre-progression” indicates the pre-progressive state of HBV-related diseases, and “Post-progression” indicates the post-progressive state of HBV-related diseases. For example, when comparisons occurred between HBV infections and CHB patients, HBV infections were “Pre-progression”, and CHB patients were “Post-progression”. As estimated by the Observed, Chao1, ACE, Shannon, Simpson, Invsimpson, and Fisher indexes, the alpha diversity reduced significantly in the disease post-progressive state (n=165) compared with the pre-progressive state (n=149).

**Figure 4:**
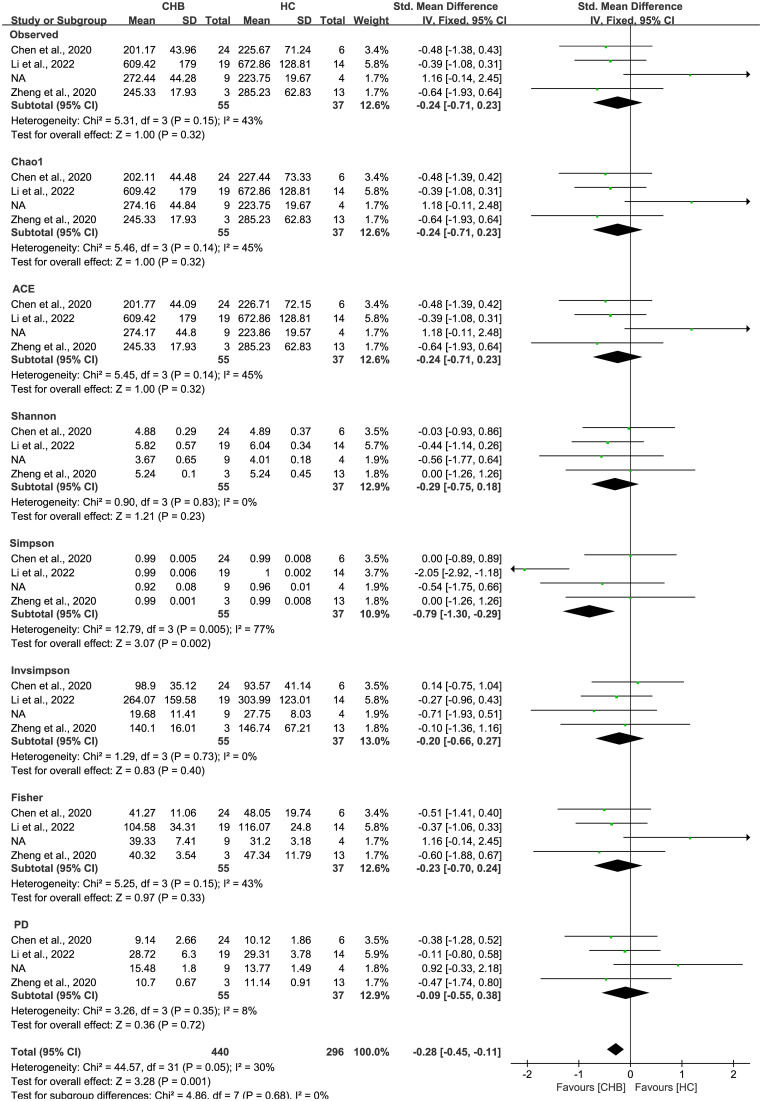
The gut microbial alpha diversity of CHB patients was decreased. The pooled outcome showed a nominate decrease in alpha diversity among the CHB patients (n=55), compared with healthy individuals (n=37). However, a numerical but no significant decrease was shown among CHB patients in each diversity index, compared with healthy controls.

**Figure 5:**
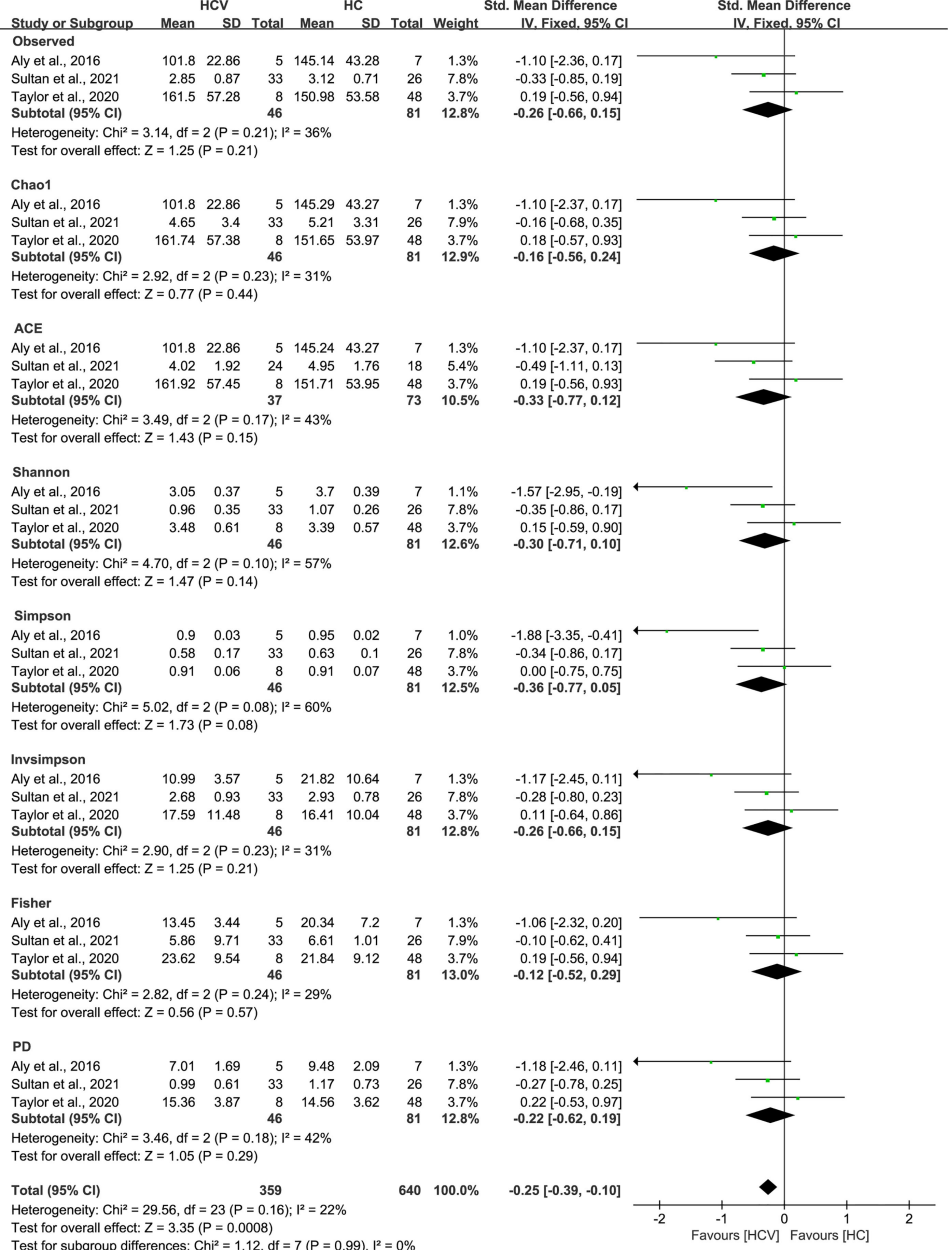
The gut microbial alpha diversity of HCV-infected patients was decreased. The pooled outcome showed a significant decrease in alpha diversity among the HCV-infected group (n=46), compared with HC individuals (n=81). However, a numerical but no significant decrease was shown among HCV-infected patients in each diversity index, compared with healthy controls.

**Figure 6:**
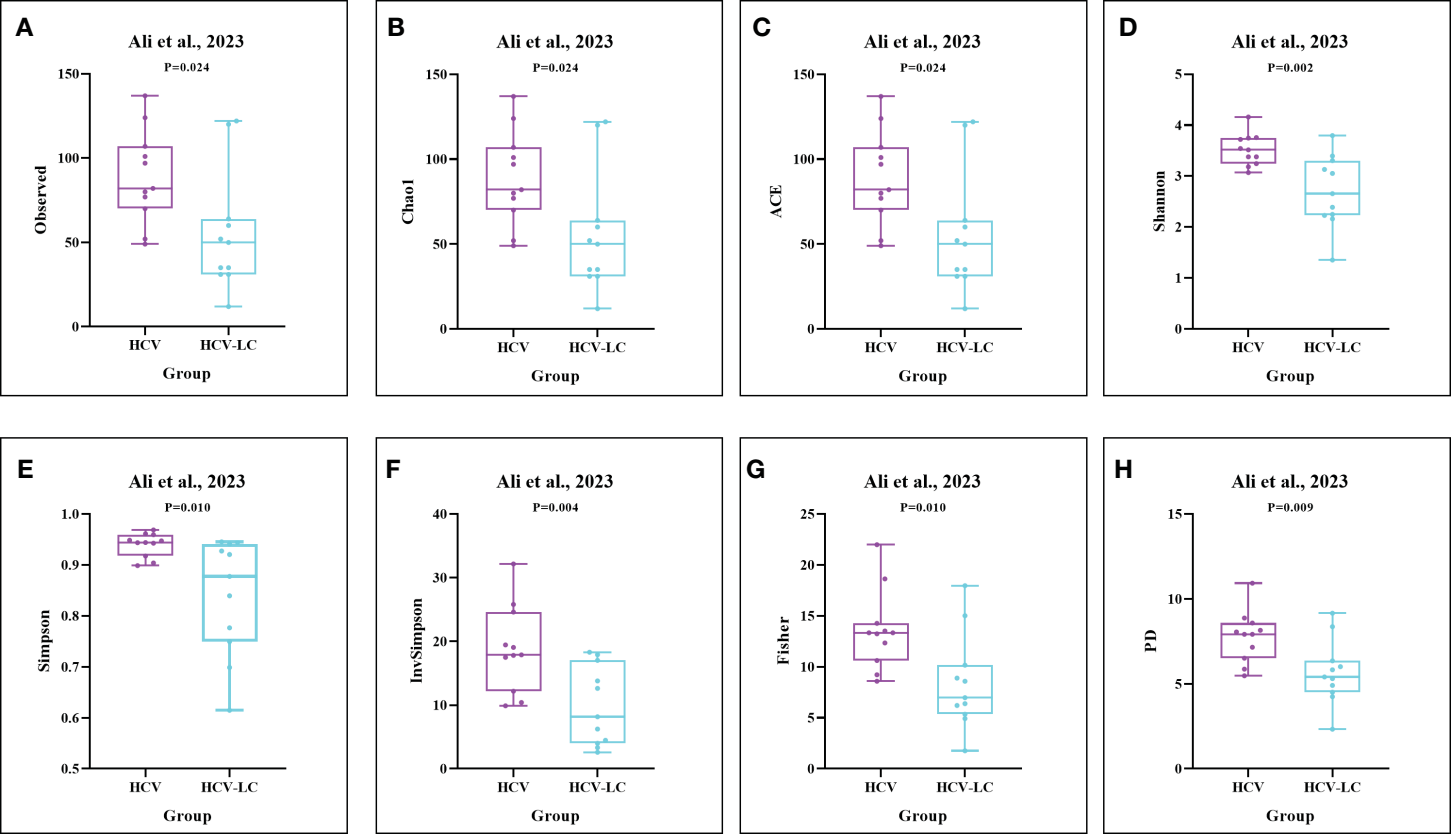
The gut microbial alpha diversity decreased as the progression of HCV infection. As estimated by the **(A)** Observed, **(B)** Chao1, **(C)** ACE, **(D)** Shannon, **(E)** Simpson, **(F)** Invsimpson, **(G)** Fisher, and **(H)** PD indexes, the results of boxplots compared between HCV-infected individuals and HCV-LC patients using the dataset of PRJNA727609 (Ali et al., 2023) showed significant decreases among HCV-LC patients (n=16), compared with HCV-infected individuals (n=13).

As estimated by the Observed Species, Chao1, Shannon, Simpson, Fisher, and PD indexes, the alpha diversity was reduced significantly in HBV-infected patients (*P* < 0.05; **Figure 2**). As HBV infection progressed, there was a significant trend for downregulation in alpha diversity according to the Observed Species, Chao1, ACE, Shannon, Simpson, Invsimpson, and Fisher indexes (*P* < 0.05; **Figure 3**). The pooled estimate showed significant decreases in chronic hepatitis B (CHB) (SMD = -0.28; 95%CI, -0.45 to -0.11; *P* < 0.05; **Figure 4**) and HCV (SMD = -0.25; 95%CI, -0.39 to -0.10; *P* < 0.05; **Figure 5**), compared with the HC group. There was a numerical but nonsignificant trend for downregulation in patients with HBV-related LC (HBV-LC) and HBV-HCC compared with the HC group (**Figures S1**, **S2**). The comparisons of the alpha diversity between groups that could not be analyzed comprehensively were shown in the form of boxplots. Compared with HCV-infected patients, the alpha diversity showed significant decreases in the (HCV-LC) group (*P* < 0.05; **Figure 6**). Meanwhile, the results of boxplots showing the changes in alpha diversity between HEV-infected individuals and patients with HEV-related acute liver failure (HEV-ALF) displayed a nonsignificant upward trend among the patients with HEV-ALF (**Figure S3**).

**Figure 7:**
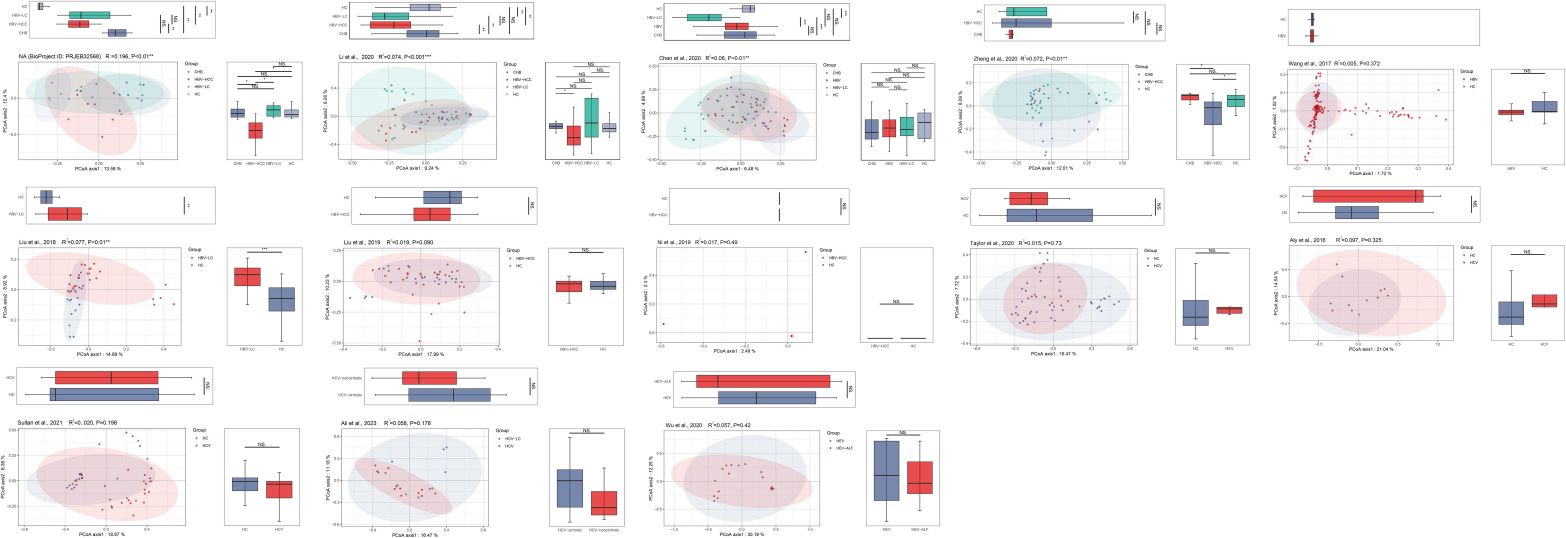
The gut microbial beta diversity changed as HBV progressed based on PCoA analysis. The PCoA analysis showed that the gut microbiome composition was significantly different based on the PERMANOVA test in the datasets of PRJEB32568, PRJNA838083 (Li et al., 2022), PRJNA558158 (Chen et al., 2020), and PRJNA540574 (Zheng et al., 2020). ^*^ *P*<0.05, ^**^ *P*<0.01, ^***^ *P*<0.001. NS, Not Statistically Significant.

**Figure 8:**
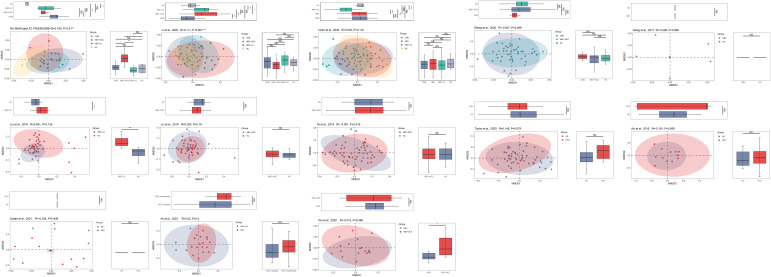
The gut microbial beta diversity changed as HBV progressed based on NMDS analysis. The NMDS analysis showed that the gut microbiome composition was significantly different based on the ANOSIM test in the datasets of PRJEB32568 and PRJNA838083 (Li et al., 2022). ^*^ *P*<0.05, ^**^ *P*<0.01, ^***^ *P*<0.001. NS, Not Statistically Significant.

The PCoA and NMDS analyses were conducted with PERMANOVA and ANOSIM, respectively, to evaluate the similarities in the gut microbiome composition among the groups in all the included studies. The PERMANOVA and ANOSIM results for the datasets of PRJEB32568 and PRJNA838083 (Li et al., 2022) reporting four separate groups (HC, CHB, HBV-LC, and HBV-HCC) indicated that as HBV infection progressed, there were significant differences in species composition between the groups (*P* < 0.01; **Figures 7**, **8**). The PERMANOVA results for the PRJNA558158 (Chen et al., 2020) and PRJNA540574 (Zheng et al., 2020) datasets showed the same trend (*P* < 0.01; **Figure 7**). Based on the ASV level, there were differences between the groups— according to the UpSet plots showing the ASV intersections between groups—including the total abundance of ASVs, the number of shared ASVs, and the number of unique ASVs for each patient group (**Figure S4**).

### 3.3 Crucial microbiota and microbial functions associated with viral hepatitis

**Figure 9:**
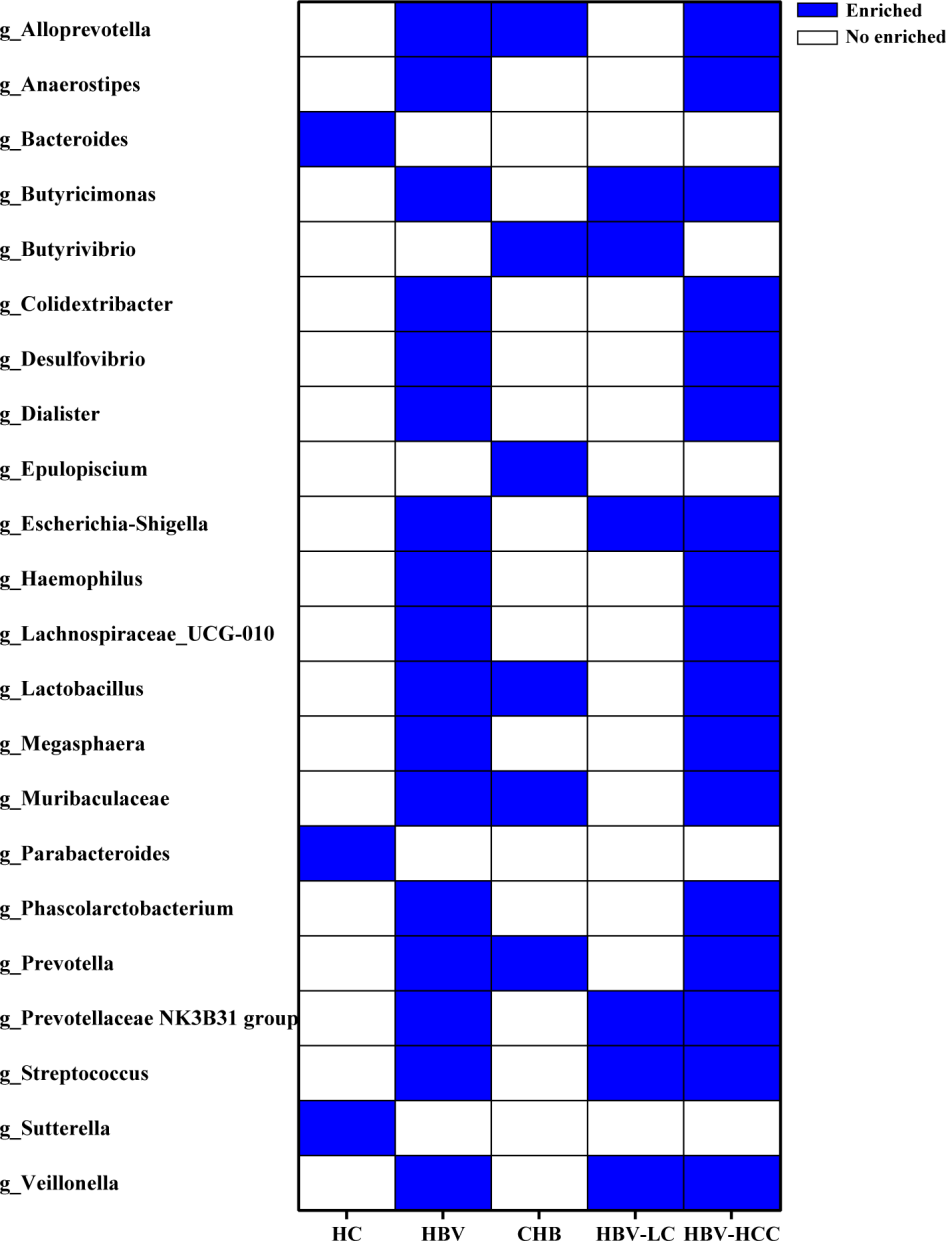
The crucial genera of the gut microbiome related to HBV infection and progression based on the results of LEfSe analysis reported by at least 2 studies. 19 genera were significantly up-regulated, while 3 genera were down-regulated in HBV-infected patients, compared with HC group.

**Figure 10:**
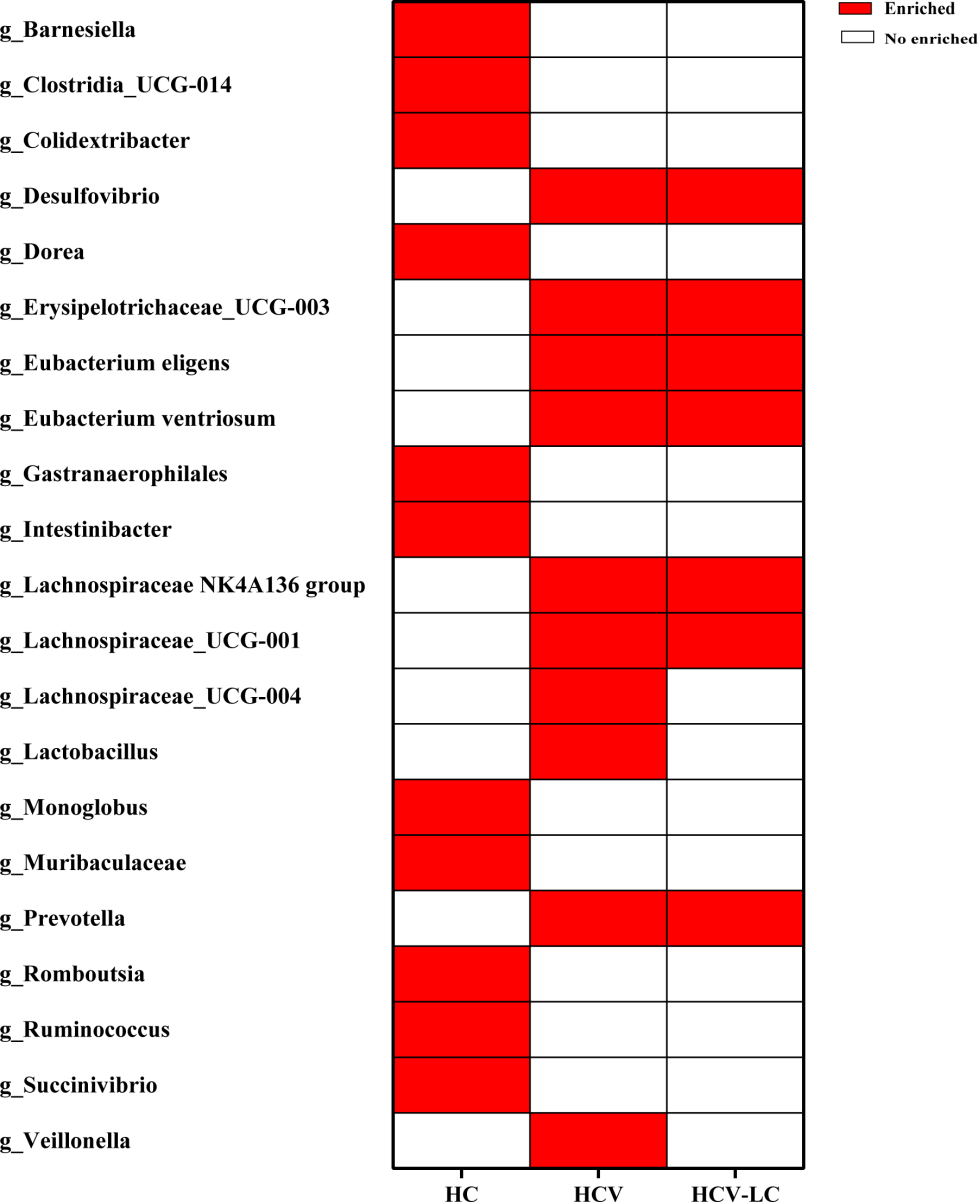
Crucial microbiome related to HCV infection and progression reported by at least 2 studies. 10 genera increased significantly, while 11 genera decreased significantly in HCV versus those in HC group.

LEfSe analysis was used to identify the dominant microbiota in each group. Based on the LDA section, the abundance of 19 genera, including *Alloprevotella*, *Butyricimonas*, and *Colidextribacter*, was significantly up-regulated in HBV-infected patients, while *Bacteroides*, *Parabacteroides*, and *Sutterella* were down-regulated, compared with those in the HC group (*P* < 0.05; **Figure 9**). Among the HCV-infected patients, 10 taxa including *Desulfovibrio*, *Eubacterium eligens*, and *Prevotalla* were increased significantly, while 11 genera including *Barnesiella*, *Colidextribacter*, and *Dorea* were decreased significantly, compared with those in the HC group (*P* < 0.05; **Figure 10**). More details of LEfSe analysis were shown in **Tables S2**-**S4**.

**Figure 11:**
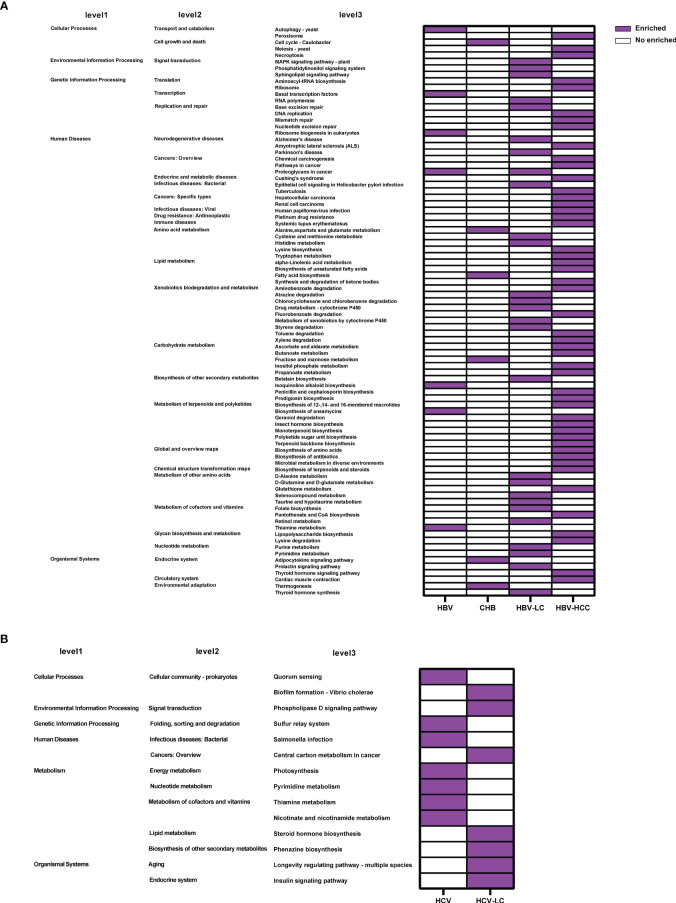
Crucial microbial functions related to **(A)** HBV or **(B)** HCV infection and progression reported by at least 2 studies. 88 microbial functions including tryptophan metabolism, fatty acid biosynthesis, and lipopolysaccharide biosynthesis were remarkably increased in HBV while 14 microbial functions including pyrimidine metabolism and thiamine metabolism were significantly enriched in HCV.

Based on 16S rRNA gene sequences, the KEGG profile was constructed to predict the microbial community function. The dominant microbial functions related to viral hepatitis were summarized in **Figure 11**. The results showed that 88 microbial functions including tryptophan metabolism, fatty acid biosynthesis, and lipopolysaccharide (LPS) biosynthesis were remarkably increased in HBV-related liver disorders (**Figure 11A**), while 14 microbial functions including lipid metabolism and thiamine metabolism were significantly enriched with HCV infection and progression (**Figure 11B**).

### 3.4 Alpha diversity and *Butyricimonas*, *Veillonella*, *Escherichia-Shigella*, and *Lactobacillus* may serve as potential gut microbial markers to predict the risk for viral hepatitis

**Figure 12:**
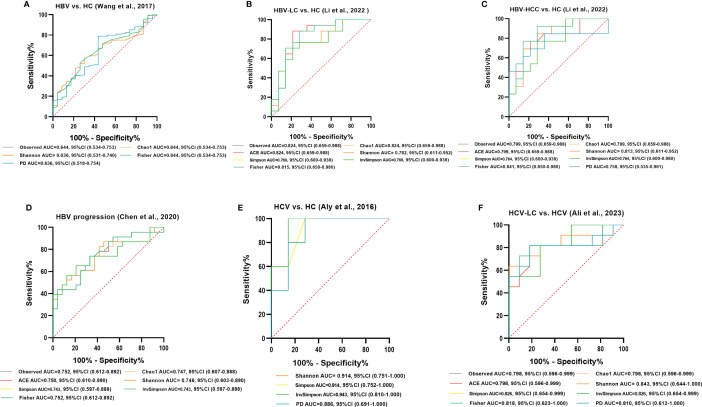
Diagnostic potential of gut microbial alpha diversity in viral hepatitis. **(A)** Five diversity indexes reached AUC values over 0.60 between HBV-infected patients (n=206) versus HC group (n=25) based on the dataset of PRJNA382861 (Wang et al., 2017). **(B)** Seven diversity indexes reached AUC values over 0.75 between HBV-LC patients (n=20) versus HC group (n=15) based on the dataset of PRJNA838083 (Li et al., 2022). **(C)** Eight diversity indexes reached AUC values over 0.75 between HBV-HCC patients (n=22) versus HC group (n=15) based on the dataset of PRJNA838083 (Li et al., 2022). **(D)** Seven diversity indexes reached AUC values over 0.70 between the post-progression state (n=53) versus the pre-progression state (n=51) of HBV progression based on the dataset of PRJNA558158 (Chen et al., 2020). **(E)** Four diversity indexes reached AUC values over 0.85 between HCV-infected individuals (n=6) versus HC group (n=8) based on the dataset of PRJNA328966 (Aly et al., 2016). **(F)** Eight diversity indexes reached AUC values over 0.75 between HCV-LC patients (n=16) versus HCV-infected patients (n=13) based on the dataset of PRJNA727609 (Ali et al., 2023).

ROC curve analysis was conducted to evaluate the potential to use the gut microbiota as a non-invasive marker to predict the risk for viral hepatitis. Those with an AUC score > 0.7 are considered to have a high risk. In the model of HBV/HCV infection and progression, all alpha diversity metrics reached AUC scores of > 0.7 (*P* < 0.05; **Figure 12**). The Observed Species, Fisher, and InvSimpson indexes reached AUC values of 0.824, 0.841, and 0.943, respectively, in the model predicting the risk for the occurrence of HBV-LC, HBV-HCC, and HCV (**Figures 12B, C, E**), and the Observed Species and Shannon indexes reached AUC values of 0.752 and 0.843, respectively, for predicting the progression of HBV and HCV (**Figures 12D, F**).

**Figure 13:**
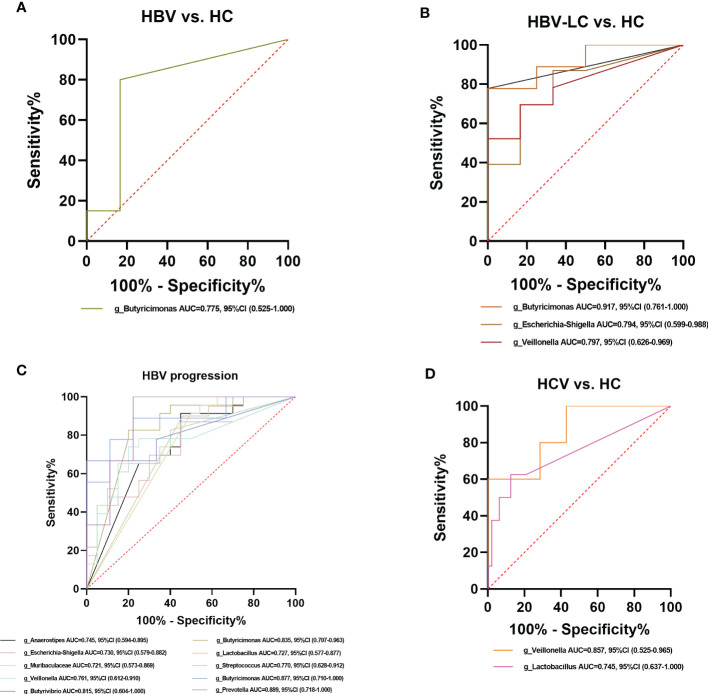
Diagnostic potential of bacteria in viral hepatitis. **(A)** The Genus of *Butyricimonas* achieved an AUC value of 0.775 between HBV-infected patients versus HC based on the dataset of PRJNA558158 (Chen et al., 2020). **(B)** Genera including *Butyricimonas*, *Butyrivibrio*, *Escherichia-Shigella*, and *Veillonella* achieved an AUC value of 0.917, 0.889, 0.794, and 0.797, respectively between HBV-LC patients versus HC group based on the dataset of PRJNA558158 (Chen et al., 2020) and PRJEB32568. **(C)** Ten genera including *Butyricimonas*, *Veillonella*, *Escherichia-Shigella*, and *Lactobacillus* had a high potential (AUC>0.7) to predict HBV progression based on the dataset of PRJNA558158 (Chen et al., 2020) and PRJEB32568. **(D)** The Genus of *Lactobaccillus* and *Veillonella* achieved an AUC value of 0.745 and 0.857 between HCV-infected patients and HC group, based on the datasets of ERP122366 (Taylor et al., 2020) and PRJNA328966 (Aly et al., 2016).

**Figure 14:**
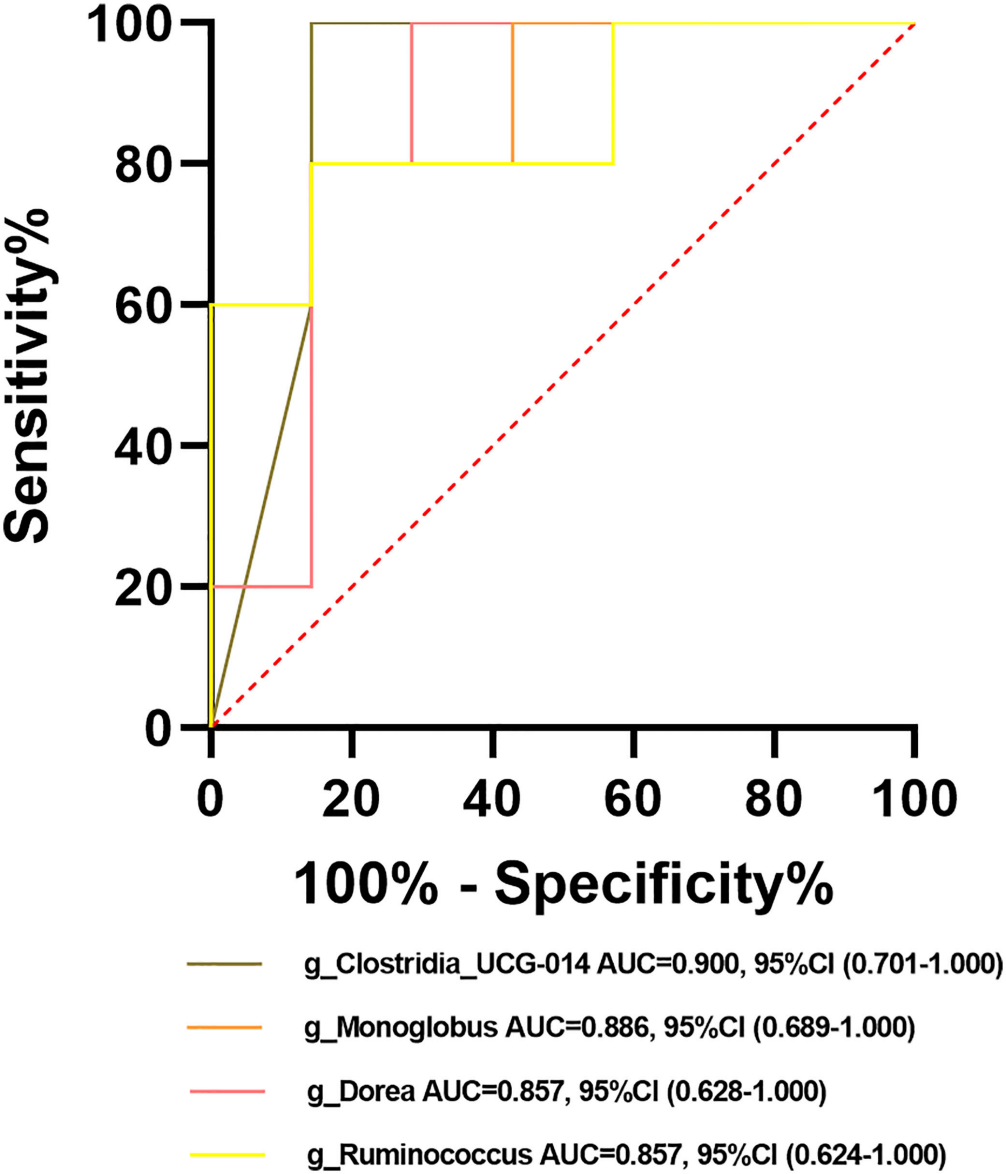
Potential beneficial bacteria in viral hepatitis. Genera including *Clostridia_UCG-014*, *Dorea*, *Monoglobus*, and *Ruminococcus* between HC and HCV achieved an AUC value of 0.900, 0.857, 0.886, and 0.857, respectively, based on the dataset of PRJNA328966 (Aly et al., 2016).

Among the crucial microbiota screened out based on the LDA section, 10 genera including *Butyricimonas*, *Veillonella*, *Escherichia-Shigella*, and *Lactobacillus* had a high potential to predict the risk of HBV progression (AUC > 0.7; **Figure 13C**). Among them, the AUC score of the particular model of *Butyricimonas* was 0.775 to predict the risk of HBV infection (**Figure 13A**). The model of *Butyricimonas*, *Veillonella*, and *Escherichia-Shigella* had AUC scores of 0.917, 0.797, and 0.794, respectively, to predict HBV-LC (**Figure 13B**). Besides, *Veillonella* and *Lactobacillus* reached AUC scores of 0.857 and 0.745, respectively, between HCV-infected patients and HC (**Figure 13D**), while genera including *Clostridia_UCG-014*, *Dorea*, *Monoglobus*, and *Ruminococcus* decreased in the HCV-infected group, with AUC values of 0.900, 0.857, 0.886, and 0.857, respectively, compared with the HC group (**Figure 14**).

## 4 Discussion

In recent years, an increasing number of studies have explored the correlation between gut microbiota perturbations and the occurrence and progression of viral hepatitis. For example, a reduction in gut microbial diversity has been linked to the severity of the disease and poor health (Wilmanski et al., 2019). Changes in the abundance of some gut bacteria can lead to the secretion of anti-inflammatory factors, thus accelerating disease progression (Peng et al., 2022). Moreover, gut microbiome-derived metabolites such as LPS and bile acids could interact with liver immune cells leading to pathological effects (Milosevic et al., 2021). However, the existing research could not identify consistent microbial taxa that respond to the disease (Wang et al., 2017; Chen et al., 2020). Few studies have systematically assessed the association between the gut microbiome and viral hepatitis, and the implication of the gut microbiota on the progression of viral hepatitis remains unclear. Therefore, we comprehensively assessed gut microbiota perturbations across a spectrum of the occurrence and progression of viral hepatitis to evaluate the reproducibility and specificity of potential gut microbial biomarkers.

Alpha diversity summarizes the structure of an ecological community regarding its richness, evenness, or both (Willis, 2019). It is a validated marker of gastrointestinal health and metabolic disorders (Plassais et al., 2021). We identified showed significant decreases in gut microbial alpha diversity after HBV/HCV infection as well as lower diversity was detected as the disease progressed, as has been observed in previous studies (Aly et al., 2016; Chen et al., 2020). This result followes the general assumption that higher microbial diversity is more beneficial to host health (Nikolova et al., 2021). Meanwhile, the AUC values of the diversity indexes were high, indicating that the change in the gut microbial alpha diversity could be a potential indicator to predict the risk for HBV/HCV infection and progression. An nonsignificant trend for upregulation in the patients with HEV-ALF versus the HEV-infected individuals demonstrated that there might be compensatory modulation in the early stage of disease progression to reestablish gut homeostasis. However, it is unclear what occurs during HAV and HDV infection due to the absence of datasets. Regarding beta diversity, we noted consistent significant dissimilarities among communities at different HBV progression stages in two datasets according to both PCoA and NMDS analyses, indicating that the gut microbiome composition was significantly alteredas HBV infection progressed. However, there were insignificant differences in beta diversity between HBV/HCV-infected patients and HC. Thus the suitability of beta diversity as a biomarker needs to be further confirmed.

LEfSe analysis showed that compared with the HC, 19 genera were dominant in HBV-infected individuals and 10 genera were dominant in HCV-infected individuals, suggesting that the changes in the abundance of crucial taxa led to significant alterations in the gut microbiome composition in viral hepatitis. Among these dominant genera, *Prevotella* could lead to a reduction in short-chain acids (SCFAs) (Iljazovic et al., 2021), which play a crucial role in delaying the progression of HBV-related HCC (Mcbrearty et al., 2021). *Lactobacillus*, *Escherichia-Shigella*, and *Veillonella* could contribute to the production of pro-inflammatory factors such as LPS and tumor necrosis factor (TNF) (Liu et al., 2022, Mata Forsberg et al., 2019). These processes may lead to the development of viral hepatitis by affecting the immune responses *in vivo* (Ji et al., 2019; Radzikowska et al., 2019; Joo et al., 2021). However, we were unable to identify the dominant microbiota related to HAV, HDV, and HEV infection due to the lack of a dataset or insufficient information provided for comprehensive analysis. At the same time, among HCV-infected individuals, we identified four decreased genera *Clostridia_UCG-014*, *Dorea*, *Monoglobus*, and *Ruminococcus* as probiotics with great potential for the prevention and treatment of HCV. Besides, we found that 10 genera, including *Butyricimonas*, *Escherichia-Shigella*, *Lactobacillus*, and *Veillonella*, could have great potential to distinguish HBV- or HCV-infected individuals from HC and to predict the risk for the development of HBV infection. The upregulation of the crucial bacteria could influence the production of gut metabolites, including tryptophan, LPS, fatty acids, and lipids (Aujoulat et al., 2014; Huang et al., 2019; Mao et al., 2020). Changes in metabolites could be associated with some microbial functions that contribute to the pathogenesis and progression of the disease (Ren et al., 2020).

In this study, functions including tryptophan metabolism, LPS biosynthesis, fatty acid biosynthesis, and lipid metabolism were significantly enriched in the HBV- or HCV-infected group. Tryptophan is an essential amino acid that possesses diverse metabolic, neurological, and immunological roles, and it is involved in viral infections including HBV (Mehraj and Routy, 2015; Fiore and Murray, 2021). A high level of LPS can promote the secretion of inflammatory cytokines including TNF and interleukin-6 (IL-6), stimulating immune cells and finally may lead to the progression of viral hepatitis (Feng et al., 2020). Fatty acids are known to play diverse roles in immune cells. It can activate the inflammatory cell signaling pathways *via* cell surface or intracellular receptors (Calder, 2011). Abnormal fatty acid levels in the liver can result in synergistic induction of HBV-related proteins and liver inflammatory factors, which might affect HBV progression (Cho et al., 2014). Lipid metabolism is intimately connected to every step of the HCV life cycle, and HCV enhances its replication by modulating lipid metabolism in the host cell (Popescu et al., 2014). All of these findings suggest that changes in specific taxa can alter the production of metabolites, which may contribute to the development of viral hepatitis through diverse microbial functions. However, few studies have been able to clarify the implication of alteration in the gut microbiome and microbial functions in viral hepatitis, which is of great significance in understanding the occurrence and progression of viral hepatitis.

This study has several limitations. First, we failed to collect all of the data from gut microbiome—related viral hepatitis studies due to the non-availability of data or inadequate information about the detailed characteristics such as grouping or disease stages. Additionally, the existing evidence limits our analysis: For example, no studies have evaluated the relationship between the gut microbiota and HAV or HDV. Finally, the authors of the included studies applied inconsistent variable regions and instruments for 16S rRNA gene sequencing, which may have generated potential bias during analysis.

## 5 Conclusions

In conclusion, our study demonstrates that the occurrence and progression of viral hepatitis are accompanied by a significant decrease in gut microbial diversity. The decreased gut microbial alpha diversity as well as the increased abundance of genera including *Butyricimonas*, *Escherichia-Shigella*, *Veillonella*, and *Lactobacillus* have the greatest potential to serve as biomarkers to predict the risk for viral hepatitis. Meanwhile, *Clostridia_UCG-014*, *Dorea*, *Monoglobus*, and *Ruminococcus* have potential value as probiotics for the prevention and treatment of viral hepatitis. Crucial microbial functions including tryptophan metabolism, fatty acid biosynthesis, LPS biosynthesis, and lipid metabolism—related to the significantly upregulated microbial community as viral hepatitis progresses—play diverse roles in the activation of immune cells and inflammatory responses. These processes provide a valuable direction to confirm the association between the gut microbiota and the occurrence and progression of viral hepatitis.

## Data availability statement

The datasets presented in this study can be found in online repositories. The names of the repository/repositories and accession number(s) can be found in the article/**Supplementary Material**.

## Author contributions

JH, HL, and PC designed the study. XinY and HM performed the study mainly including writing the manuscript, interpreting the result, and preparing the report for publication. LY supervised the process. JZ, ZL, QW, LL, FL, XipY, and BG participated in data acquisition. All authors contributed to the article and approved the submitted version.

## Supplementary material

Supplementary Figure 1 The forest plots of alpha diversity comparing HBV-LC patients to HC individuals. Results showed a numerical but no significant decrease among HBV-LC patients (n=74) in each diversity index, compared with HC group (n=41).

Supplementary Figure 2 The forest plots of alpha diversity comparing HBV-HCC patients to HC individuals. Results showed a numerical but no significant decrease among HBV-HCC patients (n=126) in each diversity index, compared with HC group (n=65).

Supplementary Figure 3 Boxplots of comparisons of gut microbial alpha diversity between HEV-infected patients and HEV-ALF groups. Results based on the dataset of ERP119119 indicated that the diversity metrics displayed a nonsignificant upward trend among the HEV-ALF patients (n=12) compared with those in the HEV-infected patients (n=12).

Supplementary Figure 4 The UpSet plots show the ASV intersections among groups in the included studies. For example, in the dataset of PRJEB32568, the total number of ASVs was 939, and 360 ASVs were shared in both groups, while 26, 105, and 43 ASVs were unique for HBV-HCC, HBV-LC, and CHB groups, respectively.
