# Supplementary figures and images for: Alterations of the gut microbiota associated with the occurrence and progression of viral hepatitis
Source: Front Cell Infect Microbiol. 2023 Jun 5;13:1119875. doi: 10.3389/fcimb.2023.1119875 (PMC10277638; doi:10.3389/fcimb.2023.1119875)

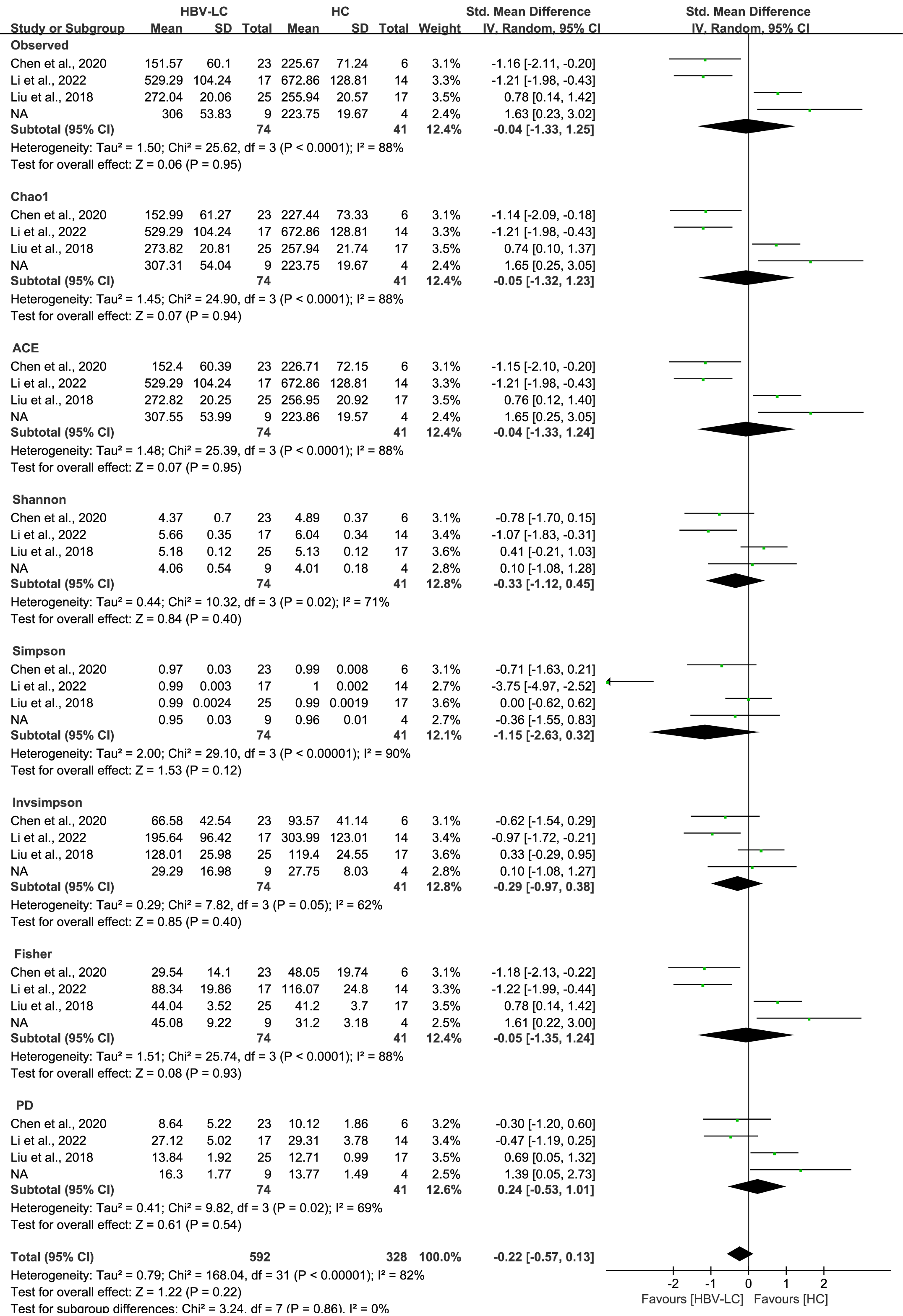

Supplement: Supplementary Figure 1 — The forest plots of alpha diversity comparing HBV-LC patients to HC individuals. Results showed a numerical but no significant decrease among HBV-LC patients (n=74) in each diversity index, compared with HC group (n=41). [file Image_1.tif]

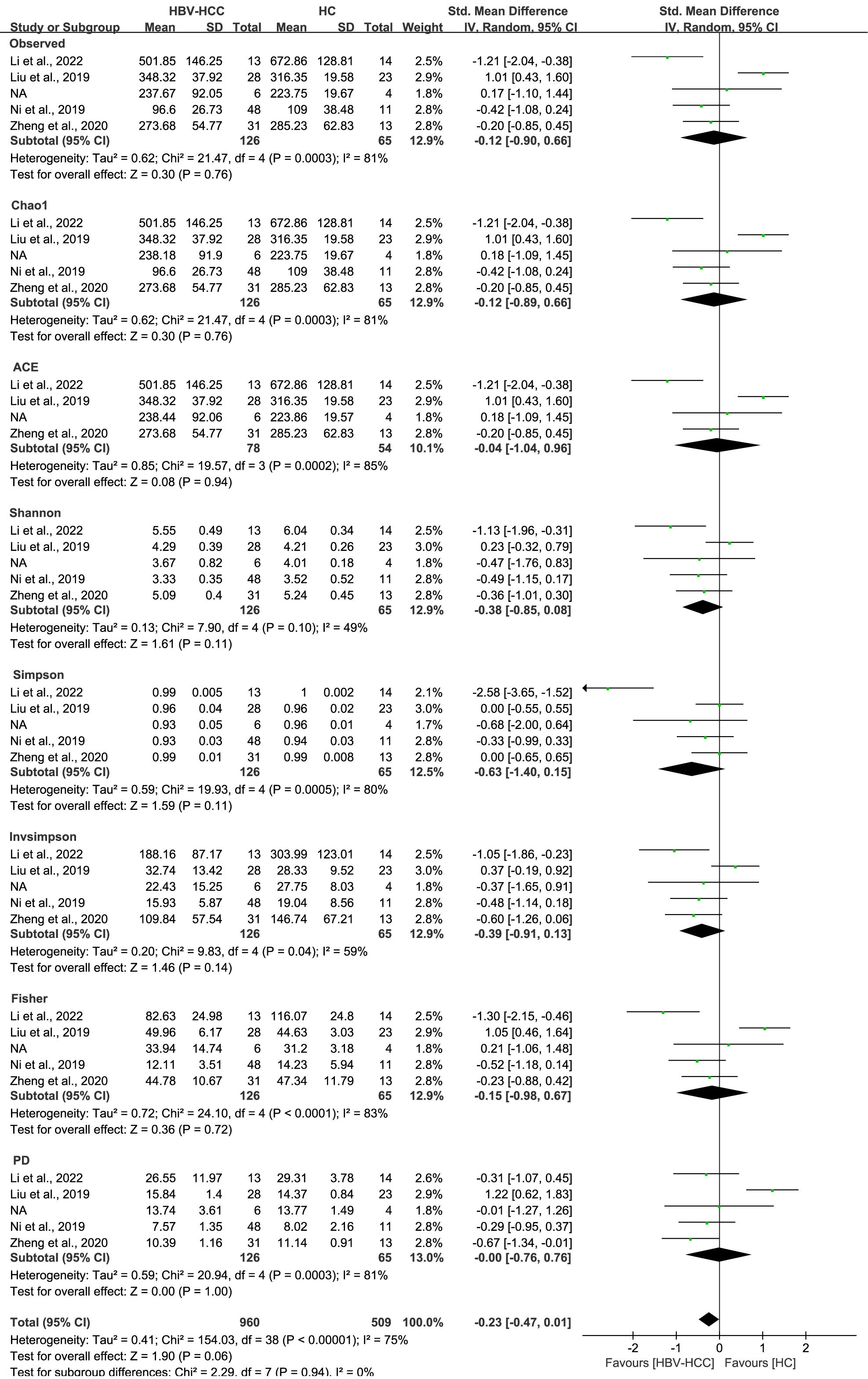

Supplement: Supplementary Figure 2 — The forest plots of alpha diversity comparing HBV-HCC patients to HC individuals. Results showed a numerical but no significant decrease among HBV-HCC patients (n=126) in each diversity index, compared with HC group (n=65). [file Image_2.tif]

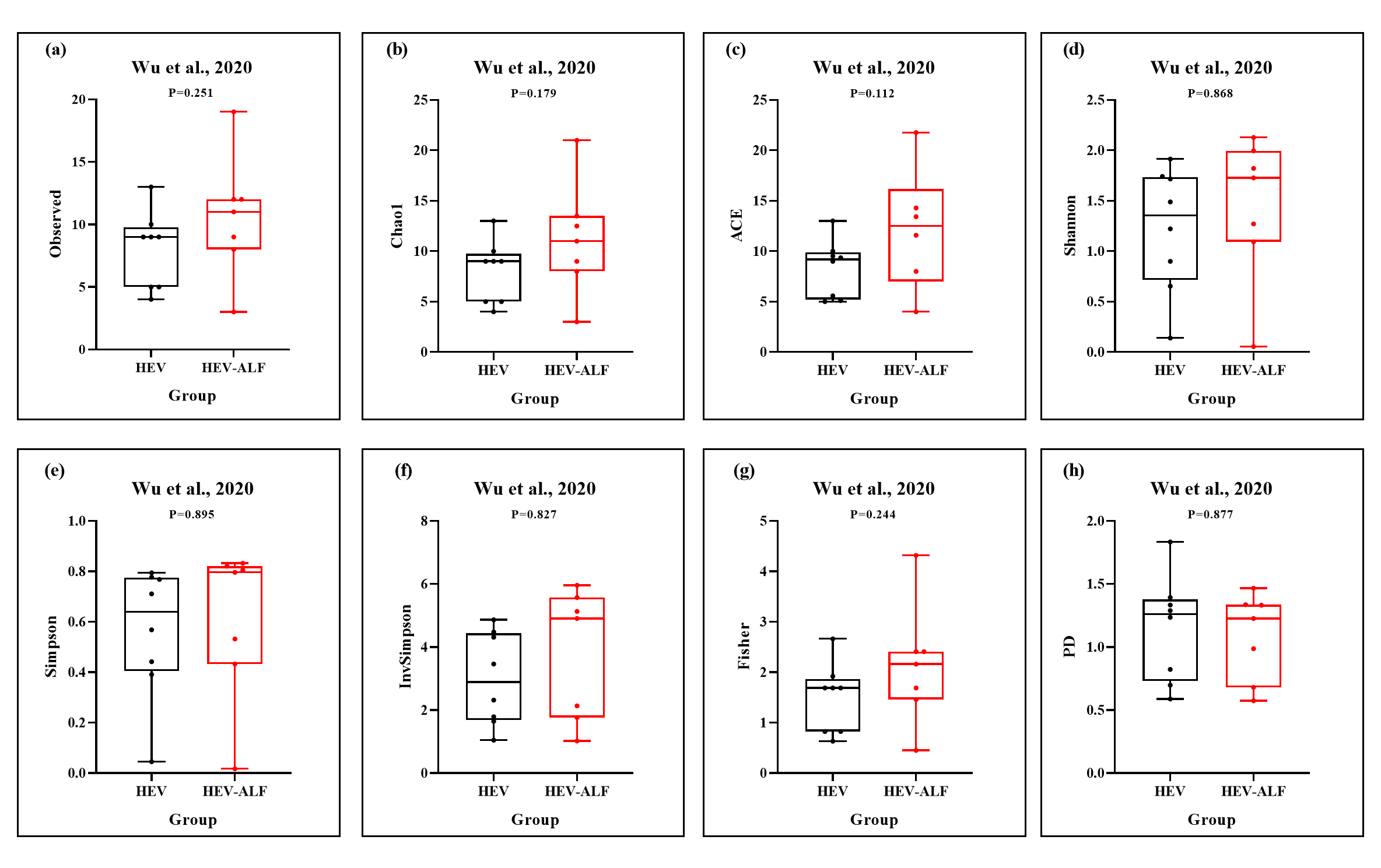

Supplement: Supplementary Figure 3 — Boxplots of comparisons of gut microbial alpha diversity between HEV-infected patients and HEV-ALF groups. Results based on the dataset of ERP119119 indicated that the diversity metrics displayed a nonsignificant upward trend among the HEV-ALF patients (n=12) compared with those in the HEV-infected patients (n=12). [file Image_3.tif]
