# Supplementary material for: Alterations of the gut microbiota associated with the occurrence and progression of viral hepatitis
Source: Front Cell Infect Microbiol. 2023 Jun 5;13:1119875. doi: 10.3389/fcimb.2023.1119875 (PMC10277638; doi:10.3389/fcimb.2023.1119875)
Supplement: Supplementary file 5 [file Table_1.docx]

| **Table S1 Search Strategy** | | |
| --- | --- | --- |
| No. | Terms | Comments |
| 1 | viral hepatitis[MeSH] OR virus hepatitis*[tw] OR hepatitis virus*[tw] | viral hepatitis |
| 2 | hepatitis A[MeSH] OR hepatitis A infection*[tw] OR hepatitis A virus*[tw] OR HAV*[tw] OR hepatitis A virus infection*[tw] OR HAV infection*[tw] OR chronic hepatitis A*[tw] OR chronic hepatitis A virus*[tw] OR chronic HAV*[tw] OR CHA*[tw] OR chronic hepatitis A virus infection*[tw] OR chronic HAV infection*[tw] | hepatitis A |
| 3 | hepatitis B[MeSH] OR hepatitis B infection*[tw] OR hepatitis B virus*[tw] OR HBV*[tw] OR hepatitis B virus infection*[tw] OR HBV infection*[tw] OR chronic hepatitis B*[tw] OR chronic hepatitis B virus*[tw] OR chronic HBV*[tw] OR CHB*[tw] OR chronic hepatitis B virus infection*[tw] OR chronic HBV infection*[tw] | hepatitis B |
| 4 | hepatitis C[MeSH] OR hepatitis C infection*[tw] OR hepatitis C virus*[tw] OR HCV*[tw] OR Hepatitis C virus infection*[tw] OR HCV infection*[tw] OR chronic hepatitis C*[tw] OR chronic hepatitis C virus*[tw] OR chronic HCV*[tw] OR CHC*[tw] OR chronic hepatitis C virus infection*[tw] OR chronic HCV infection*[tw] | hepatitis C |
| 5 | hepatitis D[MeSH] OR hepatitis D infection*[tw] OR hepatitis D virus*[tw] OR HDV*[tw] OR hepatitis D virus infection*[tw] OR HDV infection*[tw] OR chronic hepatitis D*[tw] OR chronic hepatitis D virus*[tw] OR chronic HDV*[tw] OR CHD*[tw] OR chronic hepatitis D virus infection*[tw] OR chronic HDV infection*[tw] | hepatitis D |
| 6 | hepatitis E[MeSH] OR hepatitis E infection*[tw] OR hepatitis E virus*[tw] OR HEV*[tw] OR hepatitis E virus infection*[tw] OR HEV infection*[tw] OR chronic hepatitis E*[tw] OR chronic hepatitis E virus*[tw] OR chronic HEV*[tw] OR CHE*[tw] OR chronic hepatitis E virus infection*[tw] OR chronic HEV infection*[tw] | hepatitis E |
| 7 | gut[MeSH] OR intestinal*[tw] AND microbiota[MeSH] OR microbe*[tw] OR microbiome*[tw] OR flora*[tw] OR microorganism*[tw] OR microflora*[tw] | gut microbiota |
| 8 | ((#1 OR #2 OR #3 OR #4 OR #5 OR #6) AND #7) |  |
