# Supplementary material for: Alterations of the gut microbiota associated with the occurrence and progression of viral hepatitis
Source: Front Cell Infect Microbiol. 2023 Jun 5;13:1119875. doi: 10.3389/fcimb.2023.1119875 (PMC10277638; doi:10.3389/fcimb.2023.1119875)
Supplement: Supplementary file 6 [file Table_2.docx]

| **Table S2 Crucial genera related to HBV infection and progression** | | | | |
| --- | --- | --- | --- | --- |
| Genus | Enriched group | FDR *p* | LDA | Reference |
| *g_Alloprevotella* | HBV | <0.001 | 3.436 | Wang et al., 2017 |
|  | HBV-HCC | <0.001 | 3.250 | Liu et al., 2019 |
|  | CHB | 0.032 | 3.484 | Chen et al., 2020 |
| *g_Anaerostipes* | HBV | <0.001 | 3.158 | Wang et al., 2017 |
|  | HBV-HCC | <0.001 | 2.183 | Liu et al., 2019 |
|  | HBV | 0.025 | 3.580 | Chen et al., 2020 |
| *g_Bacteroides* | HC | <0.001 | 4.289 | Wang et al., 2017 |
|  | HC | <0.001 | 4.372 | Liu et al., 2019 |
| *g_Butyricimonas* | HBV-LC | 0.021 | 3.151 | NA (PRJEB32568) |
|  | HBV | <0.001 | 3.128 | Wang et al., 2017 |
|  | HBV-HCC | <0.001 | 2.055 | Liu et al., 2019 |
|  | HBV | <0.001 | 3.786 | Chen et al., 2020 |
| *g_Butyrivibrio* | HBV-LC | 0.038 | 2.894 | NA (PRJEB32568) |
|  | HBV-HCC | 0.016 | 2.013 | Liu et al., 2019 |
|  | CHB | 0.042 | 2.956 | Chen et al., 2020 |
| *g_Colidextribacter* | HBV | <0.001 | 2.271 | Wang et al., 2017 |
|  | HBV-HCC | <0.001 | 2.129 | Liu et al., 2019 |
| *g_Desulfovibrio* | HBV | <0.001 | 2.967 | Wang et al., 2017 |
|  | HBV-HCC | <0.001 | 2.390 | Liu et al., 2019 |
| *g_Dialister* | HBV | <0.001 | 3.362 | Wang et al., 2017 |
|  | HBV-HCC | <0.001 | 3.340 | Liu et al., 2019 |
| *g_Epulopiscium* | CHB | 0.013 | 3.201 | Zheng et al., 2020 |
|  | CHB | 0.011 | 3.043 | Chen et al., 2020 |
| *g_Escherichia-Shigella* | HBV | <0.001 | 3.771 | Wang et al., 2017 |
|  | HBV-HCC | <0.001 | 3.444 | Liu et al., 2019 |
|  | HBV-LC | 0.010 | 4.718 | Chen et al., 2020 |
| *g_Haemophilus* | HBV | <0.001 | 3.291 | Wang et al., 2017 |
|  | HBV-HCC | <0.001 | 2.901 | Liu et al., 2019 |
| *g_Lachnospiraceae_UCG-010* | HBV | <0.001 | 3.221 | Wang et al., 2017 |
|  | HBV-HCC | <0.001 | 2.140 | Liu et al., 2019 |
| *g_Lactobacillus* | HBV | 0.004 | 2.216 | Wang et al., 2017 |
|  | HBV-HCC | <0.001 | 2.777 | Liu et al., 2019 |
|  | CHB | 0.006 | 3.642 | Chen et al., 2020 |
| *g_Megasphaera* | HBV | <0.001 | 3.229 | Wang et al., 2017 |
|  | HBV-HCC | <0.001 | 2.149 | Liu et al., 2019 |
|  | HBV-HCC | 0.050 | 3.639 | Zheng et al., 2020 |
| *g_Muribaculaceae* | HBV | <0.001 | 3.245 | Wang et al., 2017 |
|  | HBV-HCC | <0.001 | 2.878 | Liu et al., 2019 |
|  | CHB | 0.012 | 3.512 | Chen et al., 2020 |
| *g_Parabacteroides* | HC | <0.001 | 3.689 | Wang et al., 2017 |
|  | HC | <0.001 | 3.177 | Liu et al., 2019 |
| *g_Phascolarctobacterium* | HBV | <0.001 | 3.380 | Wang et al., 2017 |
|  | HBV-HCC | <0.001 | 3.213 | Liu et al., 2019 |
| *g_Prevotella* | CHB | 0.002 | 5.010 | NA (PRJEB32568) |
|  | HBV | <0.001 | 4.433 | Wang et al., 2017 |
|  | HBV-HCC | <0.001 | 4.121 | Liu et al., 2019 |
| *g_Prevotellaceae NK3B31 group* | HBV-LC | 0.039 | 3.681 | NA (PRJEB32568) |
|  | HBV | 0.009 | 2.704 | Wang et al., 2017 |
|  | HBV-HCC | <0.001 | 2.440 | Liu et al., 2019 |
| *g_Streptococcus* | HBV | <0.001 | 3.014 | Wang et al., 2017 |
|  | HBV-HCC | <0.001 | 2.427 | Liu et al., 2019 |
|  | HBV-LC | 0.010 | 3.885 | Chen et al., 2020 |
| *g_Sutterella* | HC | <0.001 | 3.182 | Wang et al., 2017 |
|  | HC | <0.001 | 2.928 | Liu et al., 2019 |
| *g_Veillonella* | HBV | 0.001 | 2.760 | Wang et al., 2017 |
|  | HBV-HCC | <0.001 | 2.823 | Liu et al., 2019 |
|  | HBV-LC | 0.006 | 4.286 | Chen et al., 2020 |

NA, Not Applicable.
