# Supplementary material for: Alterations of the gut microbiota associated with the occurrence and progression of viral hepatitis
Source: Front Cell Infect Microbiol. 2023 Jun 5;13:1119875. doi: 10.3389/fcimb.2023.1119875 (PMC10277638; doi:10.3389/fcimb.2023.1119875)
Supplement: Supplementary file 7 [file Table_3.docx]

| **Table S3 Crucial genera related to HCV infection and progression** | | | | |
| --- | --- | --- | --- | --- |
| Genus/Species | Enriched group | FDR *p* | LDA | Reference |
| *g_Barnesiella* | HC | <0.001 | 2.369 | Taylor et al., 2020 |
|  | HC | 0.036 | 3.210 | Aly et al., 2016 |
| *g_Clostridia_UCG-014* | HC | <0.001 | 3.926 | Taylor et al., 2020 |
|  | HC | 0.014 | 4.168 | Aly et al., 2016 |
| *g_Colidextribacter* | HC | <0.001 | 3.254 | Taylor et al., 2020 |
|  | HC | 0.009 | 2.488 | Aly et al., 2016 |
| *g_Desulfovibrio* | HCV | <0.001 | 3.021 | Taylor et al., 2020 |
|  | HCV-LC | 0.033 | 2.943 | Ali et al., 2023 |
| *g_Dorea* | HC | <0.001 | 3.334 | Taylor et al., 2020 |
|  | HC | <0.001 | 3.243 | Aly et al., 2016 |
| *g_Erysipelotrichaceae_UCG-003* | HCV | <0.001 | 3.186 | Taylor et al., 2020 |
|  | HCV-LC | 0.017 | 3.137 | Ali et al., 2023 |
| *g_Eubacterium eligens* | HCV | <0.001 | 3.029 | Taylor et al., 2020 |
|  | HCV | 0.022 | 3.461 | Aly et al., 2016 |
|  | HCV-LC | 0.025 | 3.214 | Ali et al., 2023 |
| *g_Eubacterium ventriosum* | HCV | <0.001 | 2.121 | Taylor et al., 2020 |
|  | HCV-LC | 0.025 | 3.021 | Ali et al., 2023 |
| *g_Gastranaerophilales* | HC | 0.036 | 2.423 | Taylor et al., 2020 |
|  | HC | 0.022 | 3.425 | Aly et al., 2016 |
| *g_Intestinibacter* | HC | <0.001 | 2.719 | Taylor et al., 2020 |
|  | HC | 0.036 | 2.302 | Aly et al., 2016 |
| *g_Lachnospiraceae NK4A136 group* | HCV | <0.001 | 3.645 | Taylor et al., 2020 |
|  | HCV | 0.004 | 3.344 | Aly et al., 2016 |
|  | HCV-LC | 0.033 | 2.824 | Ali et al., 2023 |
| *g_Lachnospiraceae_UCG-001* | HCV | <0.001 | 2.645 | Taylor et al., 2020 |
|  | HCV-LC | 0.033 | 3.442 | Ali et al., 2023 |
| *g_Lachnospiraceae_UCG-004* | HCV | <0.001 | 2.640 | Taylor et al., 2020 |
|  | HCV | 0.009 | 2.994 | Aly et al., 2016 |
| *g_Lactobacillus* | HCV | 0.001 | 2.877 | Taylor et al., 2020 |
|  | HCV | 0.022 | 2.948 | Aly et al., 2016 |
| *g_Monoglobus* | HC | <0.001 | 3.092 | Taylor et al., 2020 |
|  | HC | 0.006 | 3.160 | Aly et al., 2016 |
| *g_Muribaculaceae* | HC | <0.001 | 3.015 | Taylor et al., 2020 |
|  | HC | 0.014 | 3.550 | Aly et al., 2016 |
| *g_Prevotella* | HCV | <0.001 | 4.311 | Taylor et al., 2020 |
|  | HCV | <0.001 | 4.967 | Aly et al., 2016 |
|  | HCV-LC | 0.025 | 4.896 | Ali et al., 2023 |
| *g_Romboutsia* | HC | <0.001 | 3.077 | Taylor et al., 2020 |
|  | HC | 0.022 | 2.261 | Aly et al., 2016 |
| *g_Ruminococcus* | HC | <0.001 | 3.124 | Taylor et al., 2020 |
|  | HC | <0.001 | 3.054 | Aly et al., 2016 |
| *g_Succinivibrio* | HC | 0.004 | 3.168 | Taylor et al., 2020 |
|  | HC | 0.014 | 3.961 | Aly et al., 2016 |
| *g_Veillonella* | HCV | <0.001 | 2.992 | Taylor et al., 2020 |
|  | HCV | 0.014 | 3.887 | Aly et al., 2016 |
| *s_Ruminococcus bicirculans* | HC | <0.001 | 2.373 | Taylor et al., 2020 |
|  | HC | 0.006 | 2.314 | Aly et al., 2016 |
