# Supplementary material for: Alterations of the gut microbiota associated with the occurrence and progression of viral hepatitis
Source: Front Cell Infect Microbiol. 2023 Jun 5;13:1119875. doi: 10.3389/fcimb.2023.1119875 (PMC10277638; doi:10.3389/fcimb.2023.1119875)
Supplement: Supplementary file 8 [file Table_4.docx]

| **Table S4 Crucial genera related to HEV progression** | | | | |
| --- | --- | --- | --- | --- |
| Genus/Species | Enriched group | LDA | FDR *p* | Reference |
| *g__Holdemanella* | HEV-ALF | 4.463 | 0.036 | Wu et al., 2020 |
| *g__Lactobacillus* | HEV-ALF | 4.639 | 0.002 | Wu et al., 2020 |
| *g__Streptococcus* | HEV | 4.590 | 0.001 | Wu et al., 2020 |
| *g__Enterococcus* | HEV | 4.449 | 0.022 | Wu et al., 2020 |
| *g__Bacillus* | HEV | 3.221 | 0.022 | Wu et al., 2020 |
| *g__Stenotrophomonas* | HEV | 4.759 | 0.004 | Wu et al., 2020 |
| *s__Lactobacillus_mucosae* | HEV-ALF | 4.780 | 0.022 | Wu et al., 2020 |
| *s__Streptococcus_salivarius* | HEV | 4.532 | 0.002 | Wu et al., 2020 |
| *s__Bacillus_halodurans* | HEV | 3.653 | 0.036 | Wu et al., 2020 |
